# A global chronologically standardised database of high-resolution proxy sea-level reconstructions since 1800 CE

**DOI:** 10.1038/s41597-026-07232-0

**Published:** 2026-04-22

**Authors:** S. L. Williams, L. P. Jackson, E. Garrett, F. D. Hibbert, W. R. Gehrels

**Affiliations:** 1 Department of Geography, Durham University, Durham, DH1 3LE UK; 2 Department of Environment and Geography, University of York, York, YO10 5NG UK

**Keywords:** Physical oceanography, Palaeoceanography, Ocean sciences

## Abstract

Understanding relative sea-level (RSL) change since the early industrial period to the present is essential to contextualise modern trends and improve future projections. However, existing databases of Common Era RSL reconstructions contain ages derived from outdated radiocarbon calibration curves, limiting inter-comparability and integration with instrumental records. We present the Post-1800 CE Sea Level Database (P-1800SLD), a global, chronologically consistent compilation of high-resolution RSL reconstructions spanning 1800–2025 CE. The database provides a centralised dataset of ^14^C, ^210^Pb and chronostratigraphic marker data compiled from previously disparate sources. It contains 932 modelled or directly dated sea-level index points (SLIPs) from 41 sites (95% are modelled SLIPs derived from microfossil-based reconstructions) adhering to quality-based criteria. SLIP ages have been recalibrated using IntCal20 or SHCal20 and modelled using a Bayesian framework. Most updated SLIP ages agree with those originally published. Validation against tide-gauge records shows strong agreement for water levels (average r^2^ = 0.82; average RMSE = 0.10 m). Detailed meta-data are included in the database to support future updates and alternative modelling approaches.

## Background & Summary

### The role of databases in sea-level research

Databases of relative sea-level (RSL) reconstructions, which document the past position of sea level in both space and time, are crucial for ascertaining former rates, magnitudes and drivers of sea-level change. Sea-level databases are used to explore a wide range of research questions, ranging from the analysis of past sea-level trends at a local scale^1^, to reconstructions of basin-scale and global sea-level change, and the attribution of sea-level drivers through time^2^. Palaeo sea-level reconstructions also help to provide context for modern and projected future rates of sea-level change and are essential to improve our understanding of spatio-temporal changes. Furthermore, reconstructions within databases provide an independent means of validating models of sea-level change and their components (e.g., glacio-isostatic adjustment models^1^), which in turn improves the closure of regional sea-level budgets^2^. Finally, palaeo sea-level databases also offer opportunities to assess ecosystem vulnerability (e.g., salt marsh/coral drowning^3^) and socio-economic adaptation responses^4^.

Recent efforts have led to the creation of sea-level databases for a range of geological periods. These include: the Last Interglacial^5^, Last Glacial Maximum^6,7^, Holocene^7,8^ and Common Era^2,9,10^ – a comprehensive list of regional-scale databases is described in Khan *et al*.^7^. Despite this progress, research questions remain, particularly for recent sea-level change (i.e., the Common Era, the last 2000 years), owing to uncertainties in the temporal and vertical resolution of indicators and associated reconstructions^11,12^, inter-comparability between sites^12^, and interpretability of the reconstructed sea-level signals when compared to modern instrumental records. For example, can proxy records serve as geological tide gauges to refine regional sea-level budgets where instrumental data are lacking^13^? Might they be used to understand sub-decadal variability in the ice-ocean response to short-term, internal climate forcing^13^? Can they constrain future sea-level projections^14^? And, do they indicate regional variability in the departure from the Holocene background rate following the Industrial Period^15^? The database constructed here allows users to directly address these questions, enabling robust investigation of sea-level change since 1800 CE in concert with instrumental observations.

### Sea-level indicators and directly dated versus modelled sea-level index points

**Fig. 1:**
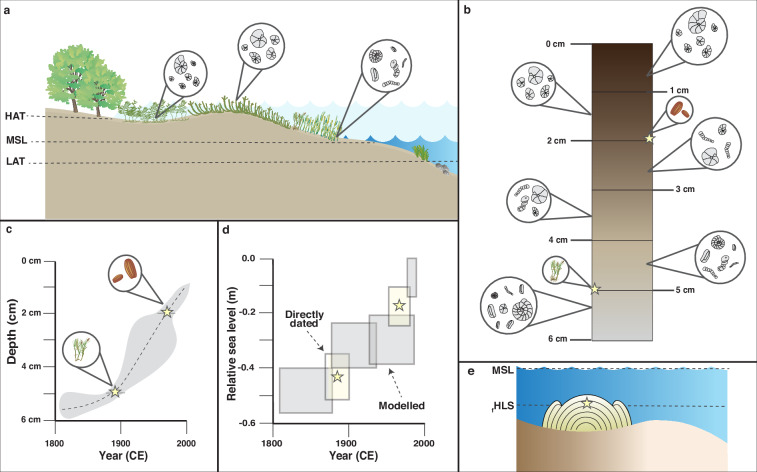
Schematic of sea-level index point generation from directly dated and modelled sea-level index points. (**a**) Conceptual cross-section of a transect across a salt marsh from the upland to the subtidal zones, showing tidal datums—Highest Astronomical Tide (HAT), Mean Sea Level (MSL), and Lowest Astronomical Tide (LAT)—and example distribution of foraminifera (sea-level indicators) in the modern environment. (**b**) Sediment core with continuous fossil foraminiferal assemblage sampling and two radiocarbon (^14^C) samples. (**c**) Age–depth model constructed from discontinuous ^14^C samples obtained from the sediment core to produce continuous ages for every centimeter of the sediment core. (**d**) Relative sea-level index points derived from directly dated and modelled ages. (**e**) Schematic illustrating a coral microatoll (sea-level indicator) showing present MSL and former Highest Level of Survival (_f_HSL) indicated by the diedown. Some icons are adapted from the Integration and Application Network, University of Maryland Center for Environmental Science (ian.umces.edu/media-library)^45^.

A sea-level indicator is a physical sample or feature that was formed in relation to a past sea level (e.g., a tidal notch, coral microatoll *etc;* Fig. 1a). A sea-level index point (SLIP) defines the discrete vertical position of a past RSL in time and space (as opposed to marine or terrestrial limiting points, which only provide a constraint on sea level i.e., indicating that it was above or below a certain elevation)^16^. A SLIP is established using specific criteria to ensure it accurately represents past sea level. Each SLIP must have: a clearly defined location, the elevation of the sea-level indicator corrected for a known relationship between the indicator and a contemporary tidal level (referred to as the indicative meaning)^16–18^, and a calibrated age typically obtained through radiometric dating (e.g., ^14^C) or relative dating (*e.g*. annual band/layer counting) with their associated uncertainties.

SLIP elevation uncertainty (known as the indicative range) varies by indicator type and the local environmental context. Modern analogue methods typically employed produce SLIP elevation uncertainty on the order of metres to decimetres, though samples such as coral microatolls and salt marsh microfossils can yield centimetre-scale ranges^7^. In the case of sedimentary environments (e.g., salt marshes or mangroves), multiple SLIPs and their uncertainties are calculated at depths in a core that contain sufficient abundances of microfossils^11,19^ (Fig. 1b) such as diatoms or foraminifera by applying a transfer function that defines the relationship between the modern distribution of microorganisms and tidal elevation^20^. In some locations, sediment compaction must be considered when determining SLIP elevations, as it can lead to mis-estimation of sea-level rates^21^.

SLIP ages can be characterised in two ways: first via direct dating of the sea-level indicator followed by calibration (if required) i.e., *a directly dated SLIP* (Fig. 1b), and second via interpolation of a modelled age-depth relationship (derived from directly dated core samples) at the depth of the sea-level indicator i.e., *a modelled SLIP* (Fig. 1c,d) (see Parnell and Gehrels^22^ for definitions and further explanation). The second approach is used widely in high-resolution reconstructions where it is often impractical to obtain large numbers of direct dates. During the age-depth modelling process, directly dated sea-level indicators from the core will have their ages replaced by the modelled ones. Although some studies do not distinguish between modelled SLIPs and ‘true’ SLIPs e.g.^23–26^, we refer to RSL estimates with an age derived from an age-depth model as a modelled SLIP following Parnell and Gehrels^22^.

Generally, temporal uncertainty of a directly dated SLIP is often tens to hundreds of years, though annual growth lines (e.g., in coral microatolls) can yield high-precision chronologies^27^ (Fig. 1e). Series of modelled SLIPs from single sediment cores often achieve higher temporal resolution (e.g., 5–20 years), particularly when multiple chronostratigraphic markers are used in the age-depth model. Post 1800 CE, there are usually improvements in resolution and uncertainty due to abundant anthropogenic markers^24^. It is important to highlight the difference between SLIP age determination as defined above, as the age-modelling approach has the potential for differences in ages and their uncertainties depending on the modelled relationship used^28^. In this study we identify the SLIP age characterisation of each sea-level indicator to allow an update of ages following new calibration curve release or the use of alternative age-depth models to facilitate intercomparison and chronologically consistent global sea-level analyses.

### The importance of standardised and open data for indicator-instrument harmonisation

Data standardisation is integral to enabling consistent and unbiased analyses and comparisons between palaeo sea-level data from disparate locations with instrumental sea-level data. The need for standardisation arises, in part, from the range of methodological choices that can be applied during the process of palaeo sea-level reconstruction and influence spatial and temporal aspects of the result. Decisions made during data collection in the field, laboratory analysis and statistical modelling all influence a sea-level reconstruction. These are explored below for series of modelled SLIPs from single sediment cores, which form the majority (39 sites; 95%) of the sea-level data in this database.

When establishing estimates of RSL using microfossils (e.g., foraminifera or diatoms) from single cores, many choices are made, including: training set size and composition^19^, total count in a fossil assemblage^11^, choice of transfer function (*i.e*. Traditional – weighted averaging, partial least squares *etc* versus Bayesian^20,29^), choice of age-depth model^30^, inclusion/exclusion of compaction effects^21^, and, should a researcher seek to interpolate between SLIPs, the choice of statistical model used for inference^31^. Indeed, there are valid reasons why a researcher may take various decisions for different reconstructions, such as addressing taphonomic bias in fossil assemblages, considering the effect of inclusion or exclusion of natural variability within a training set, or attempting to reduce vertical error whilst considering precision versus accuracy (e.g., removal–or not–of outliers to reduce root-mean-squared error). All these decisions lead to subtle, but important, differences in relative sea-level reconstructions.

High-resolution Common Era reconstructions with RSL estimates in the 19^th^ and 20^th^ centuries are often compared with observational data, typically nearby tide-gauge data, for calibration and verification^32^. While tide-gauge data are temporally consistent, databases of proxy data are not, as different sites often employ a variety of age-depth modelling approaches. This limits direct comparison between data types, preventing their joint usage. Research by Blaauw *et al*.^33^ has demonstrated the advantage of Bayesian age-depth models over classical ones and most, if not all, Common Era sea-level reconstructions which employ an age-depth model now utilise one of three frameworks: rplum/rbacon^34,35^, Bchron^36^ or OxCal^37^. There are sound reasons for employing one model over another, and these decisions are often site dependent. Bchron allows users to take depth uncertainty into account^38^, which is particularly useful where a chronostratigraphic boundary occurs over several centimetres, or where, for example, rhizomes are dated^10^. Rplum/rbacon enables the integration of multiple dating methods within one statistical framework, which is useful for post-industrial reconstructions over the last 200 years where the radiocarbon plateau limits the precision of ^14^C dates and alternative isotopic dating methods/chronohorizons featuring distinct ecological or geochemical signatures can be utilised to provide empirical constraint. For example, in rplum, users can input both raw ^210^Pb and ^14^C data into a single model. The amalgamation of multiple dating methods often increases accuracy and improves precision of the resulting output. Another key advantage of using rplum/rbacon is the ability to directly model post-bomb ^14^C ages without requiring prior conversion to calendar ages in, for example, CALIBomb. OxCal is advantageous for new users, owing to its thorough documentation and supportive online community. Additionally, researchers can run models through a graphical user interface (Bchron and rplum/rbacon must be coded in statistical software R).

However, whilst these models generally produce similar mean age estimates, age uncertainty can vary substantially^39^. For example, Traschel and Telford^39^ conducted an experiment using sediment with a known age-depth relationship (dated by varve counting). They showed that Bchron produced ensembles where more than the expected 66.7% of true ages fell between the 16.6% and 83.3% quantile bounds, indicating the model created was overly cautious. In OxCal, varying the k parameter (which affects the rigidity/ flexibility of the model) systematically influenced uncertainty estimates, with high k values producing overly narrow (overconfident) ensembles that underestimated uncertainty, and very low k values resulting in overly wide (pessimistic) ensembles. This poses a challenge for comparing proxy and observational sea-level records due to possible shifts in phase where the onset of RSL changes is important to characterise.

Improvements to the radiocarbon calibration curves^40,41^ since 2020, driven by the availability of new data and advancements in statistical modelling, mean that radiocarbon ages of reconstructions published prior to 2020 should be updated to facilitate internal consistency between sites. These updates enhance the accuracy and precision of subsequent relative sea-level reconstructions, thereby increasing confidence when comparing them to observational data or regional sea-level budgets. Previous Common Era databases^2,9,10^ contain calibrated modelled ages derived from now outdated calibration curves, rather than raw radiocarbon data. As such, they contain a mixture of ages based on different calibration models, introducing inconsistency and limiting comparability across sites. Post-1800 CE, differences in calibration vary by up to 50 years, particularly for radiocarbon ages pre-1940 and in the Northern Hemisphere. This can be important when trying to assess interannual variability in sea level as decadal-scale systematic misalignments may offset events. The latest calibration curves and previous iterations can be viewed and downloaded from https://intcal.org/curves.html.

The atmospheric radiocarbon curve for the period 1950–2019 (the “bomb series”) has also been updated through successive compilations from Bomb04 to Bomb13, and most recently to Bomb21^42^. Bomb21 incorporates substantially more data (22 tree-ring ¹⁴C series, compared to 11 in Bomb13) and uses a curve-fitting approach that smooths individual measurement uncertainties rather than the weighted-averaging method used in Bomb13. These revisions extend the curve both prior to 1950 and beyond 2013 and produce smoother, less noisy records across all zones (c.f. Fig. 6 in Hua *et al*.^42^). Bomb13 originally combined monthly data (where measurements existed) with annual summer averages elsewhere, resulting in mixed temporal resolution; Bomb21 now has consistent monthly data. Compared with Bomb13, the updated F^14^C values in Bomb21 are generally slightly lower across all zones. By adopting Bomb21, reconstructions of recent radiocarbon ages benefit from improved precision, reduced noise, consistent temporal coverage and better representation of seasonal cycles. Consequently, maintaining a database of raw radiocarbon measurements that can be periodically updated is essential for ensuring consistency and comparability across sites. Further methodological details and the latest datasets are provided in Hua *et al*.^42^.

To date, no Common Era sea-level database has systematically provided the full suite of raw data required to update ages of sea-level reconstructions. Information necessary for updating and revising age-depth models is currently distributed inconsistently across the literature: some studies provide all data required for model updates, others provide partial information, and in some cases the relevant data are not publicly available and must be obtained directly from the original authors. These inconsistencies largely reflect evolving norms in data archiving and reporting, with expectations for open data and adherence to FAIR principles^43^ becoming more formalised over recent years. The HOLSEA database^7^ and the more recent release of the HOLSEA Datahub^44^ are excellent examples of the utility and power of community-led efforts to facilitate open data.

As such, here, we present the first comprehensive, open database of raw chronological data which underpin post-1800 CE proxy-based sea-level reconstructions^45^. The database includes available uncalibrated ^14^C measurements, together with radiocarbon laboratory IDs, material dated, appropriate calibration and post-bomb calibration curves and any fraction modern ^14^C data. It also provides raw ^210^Pb data (including bulk density estimates) and the year in which these measurements were made. Chronostratigraphic marker data are provided, including thickness uncertainties where markers span multiple centimetres of a core. The database also includes all depths of palaeomarsh estimates used to generate RSL estimates in the publication, enabling users to identify the precise depths for which an age needs to be extracted. Finally, where site location information was absent from the original publications, geographic coordinates have been newly compiled and provided. This database enables direct comparisons between sites, facilitates overlap with instrumental data, and is in phase with the longest historical ocean and climate reanalysis models (e.g., SODA^46^, CMIP^47,48^).

While the selection of a transfer function to derive palaeo sea-level estimates in space is a critical factor in sea-level reconstructions which also affects internal consistency, as previously highlighted, it is highly complex and site dependent due to uncertainties with applying appropriate reference water levels^49^, comparisons with local water-level data, and site-specific environmental factors that influence microfaunal distributions and resulting transfer functions^50^. Uniform implementation of recent methods like the Bayesian Transfer Function^51^ also may not be possible for some sites based on highly diverse modern training sets (e.g., diatom-based studies with hundreds of species) given the computational power required. Nonetheless, these further steps in standardisation may be possible in future research, but they are beyond the scope of this paper. As such, this database should not be viewed as a final resource, but rather as an initial step towards open data practices and a centralised repository for future updates.

### Database summary

We implemented a structured sifting and classification workflow to: (1) identify high-resolution sea-level reconstructions and (2) undertake recalibration and age-depth modelling, where necessary, to generate an updated, chronologically standardised sea-level database. To ensure a common reference period with instrumental observations, we further required that reconstructions contain at least four sea-level index points (SLIPs) between 1950 and 2000 CE. We restrict the processed dataset to sites spanning 1800–2025 CE to facilitate analyses of decadal to multidecadal sea-level variability for a time period with high societal relevance, however input data to the age-depth models utilises all available chronological data (i.e., pre-1800 CE). This interval enables greatest chronological precision and reduced age uncertainty, avoiding the temporal hiatuses and larger dating uncertainties that commonly affect pre-1800 records. All available raw age data for each processed site are provided in separate tables, enabling users to interrogate every individual record if desired. Detailed descriptions of these procedures are provided in the Methods.

**Fig. 2:**
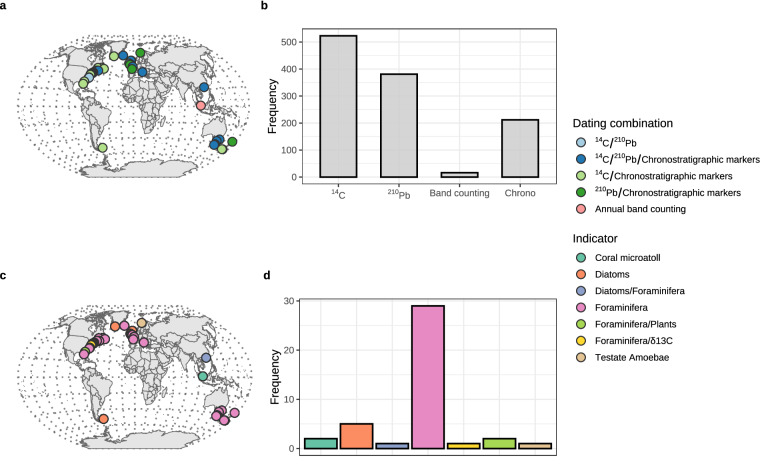
(**a**) Chronological data types (i.e., availability for each site in P1800-SLD^45^), (**b**) frequency of chronological data type, (**c**) indicator type for each site and (**d**) frequency of indicator type.

The database is comprised of 41 sites (39 from sedimentary archives and 2 from individual coral microatolls) and 932 SLIPs (95% modelled, 5% directly dated). The database, herein referred to as the Post-1800 CE Sea Level Database (P-1800SLD^45^) is global in scale, though there is a bias to the Northern Hemisphere (n = 33) compared to the Southern Hemisphere (n = 8) (Table 1; Fig. 2). There is large variability in the combination of dating approaches to generate ages for RSL estimates. Only two records use a single dating method approach (annual band counting) and so generally, a multi-method approach is used to construct a site’s age-depth model (Fig. 2a). This is unsurprising given the temporal resolution needed for investigations of sub-decadal sea-level change. There is broad geographic variability in which methods are employed to generate age estimates, with some studies utilising ^14^C and ^210^Pb, some ^14^C and chronostratigraphic markers, some ^210^Pb and chronostratigraphic markers and some all three (Fig. 2a). This suggests that use of a particular dating method is likely driven by site-specific factors and available resources.

**Table 1:** Source references for sites included in the P-1800SLD^45^.

Region	Sites (with References)
Norway	Storosen^23^
Iceland	Víðarhólmi^69,70^
Greenland	Dronning Marie Dal^13^
Scotland	Kyle of Tongue; Loch Laxford^59^
Ireland	Dublin^32^; Bracky Bridge^71^
Wales	Malltraeth^72^
UK	Newtown Marsh^60^
Newfoundland, Canada	Placentia; Big River Marsh^10^
Quebec, Canada	Saint-Siméon^73^
France	Morbihan Golfe^74^
Maine, USA	Sanborn Cove^75–77^
Croatia	Jadrtovac^78^
Massachusetts, USA	Belle Isle^79^
Nova Scotia, Canada	Chezzetcook^75,80^
Rhode Island, USA	Fox Hill^31^
Connecticut, USA	East River Marsh^31,81^; Barn Island^75,82^
New York, USA	Pelham Bay^31,83^
New Jersey, USA	Cape May Courthouse^29,84^; Cheesequake^2^; Barnegat Bay^85^
North Carolina, USA	Sand Point; Cedar IslandTump Point^62,63^; Salvo^64^
Florida, USA	Little Manatee River^86^; Nassau^87^
China	Pearl River^88^
Indonesia	Mapur^27^
Australia	Lutregala; Little Swanport; Tarra; Wapengo^24,89^
New Zealand	Mokomoko; Pounawea; Puhinui^90–92^
Falkland Islands (Islas Malvinas)	Swan Inlet^25,61^

Overall, ^14^C (n = 523) is the most frequently employed method to date sediment cores (Fig. 2b). Among dated material, plant macrofossils are predominant (n = 458), followed by bulk sediment (n = 63), while insect carapaces have only been used in three cases, one of which is in combination with a plant macrofossil. Plant macrofossils are advantageous over bulk sediment as they typically yield tighter chronological constraint than bulk sediment, as sediment contains a mixture of *in-situ* and *ex-situ* carbon sources which can complicate and obscure true ^14^C ages. Where available, the database documents the type of macrofossil dated, for example distinguishing between rhizomes and detrital plant material. Dating rhizomes may introduce bias if the material is younger than the targeted horizon, while detrital material may have been subject to reworking. In both approaches, such risks are minimised through careful observation and sample handling. There are 381 ^210^Pb and associated bulk density measurements and 212 chronostratigraphic markers (i.e., known dates derived from documented events, typically using exotic pollen, trace metals and isotopes ^137^Cs and ^206^Pb/^207^Pb). Annual band counting was used to provide dates for 16 RSL estimates from 2 coral microatoll records (Fig. 2b).

The use of foraminifera as sea-level indicators is ubiquitous around the world, but they are used near-exclusively in the Indo-Pacific (at all sites bar Swan Inlet, Falkland Islands (Islas Malvinas) and Mapur, Indonesia) (Fig. 2c). There is also prevalent use of foraminifera in the North Atlantic and diatoms and testate amoebae at high latitudes in both hemispheres (Fig. 2c). Sea-level indicators include foraminifera, foraminifera with a secondary indicator i.e., plants, *δ*^13^C, diatoms, coral microatolls and testate amoebae. Foraminifera are the most employed indicator, used in 29 reconstructions independently, and in a further four alongside other proxies (Fig. 2d). This is followed by diatoms (n = 5 independently; 1 alongside foraminifera).

**Fig. 3:**
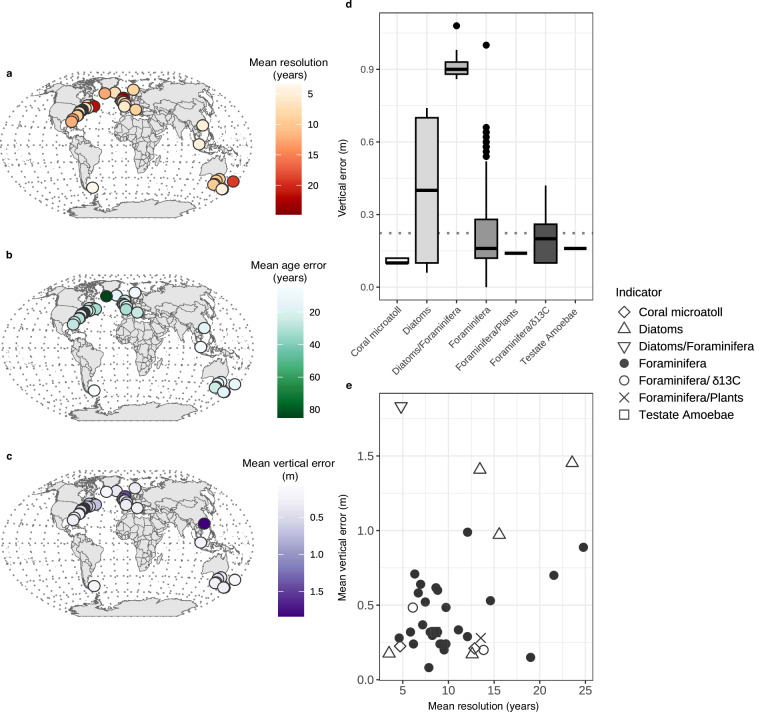
(**a**) Updated mean record resolution (based off central age estimates), (**b**) updated mean age error, (**c**) mean vertical error, (**d**) vertical error associated with each indicator – grey dotted line shows mean vertical error (errors given to 2σ) and (**e**) relationship between mean vertical error and mean resolution^45^.

The average temporal resolution (using central age estimates) ranges from 4 years per SLIP (Swan Inlet) to 25 years per SLIP (Cheesequake, New Jersey) and the mean average resolution across the database is 9 years per SLIP (Fig. 3a). Minimum age error ranges from less than a year to a maximum of ±112 years (2σ; Dronning Marie Dal, Greenland). Across the database, the average mean age error is ±19 years (2σ). Excluding one SLIP from Puhinui, New Zealand with zero error, mean vertical error ranges from 0.04–0.92 m (2σ), with the full observed range – from the lowest vertical error to the highest – spanning 0.04–1.08 m (2σ). Across the entire database, the average vertical error is 0.22 m (2σ; Fig. 3c). The sea-level reconstructions with the average lowest age and vertical error are derived from coral microatolls (average 2.25 years; 0.11 m (2σ)), however n = 2 (Fig. 3d). Generally, diatoms, as sea-level indicators, result in sea-level reconstructions with the largest average vertical uncertainty (mean 0.38 m) (2σ), but also a large range (0.06–0.74 m (2σ); Fig. 3d), with the smallest vertical error from the reconstruction at Dronning Marie Dal, Greenland and the largest at Loch Laxford, Scotland when used independently. When incorporated with foraminifera, this upper range increases to 0.92 m (0.86–1.08 m) (2σ) at Pearl River, China. Reconstructions derived from foraminifera independently have an average vertical uncertainty of 0.20 m, though ranges can vary from ~0.4 m to 1 m (2σ; Fig. 3d). As such, they have the largest range of all indicators.

Sea-level indicators clearly exhibit inherent differences in vertical precision. For example, coral microatolls are physically constrained by tidal elevation, ceasing growth when subaerially exposed; this restricts them to a very narrow vertical range and provides high precision as sea-level indicators. In contrast, certain microfossil species, such as diatoms, can tolerate a wide range of salinities from saline to brackish conditions, allowing them to occupy a broader portion of the intertidal zone and resulting in comparatively larger vertical uncertainties. Indicators in the Indo-Pacific tend to have the lowest vertical error and highest temporal resolution, whilst those in the North Atlantic tend to have the largest vertical error and lowest resolution (Fig. 3e). This pattern is typical as vertical uncertainty is largely related to the indicative range of the indicator, which is predominantly influenced by tidal range, with sites with larger tidal ranges generally having greater vertical uncertainty^50^. Thus, the overall precision of a sea-level reconstruction is both a function of indicator type and tidal range, with indicators with limited elevational range located in microtidal regimes often yielding the highest precision reconstructions.

**Fig. 4:**
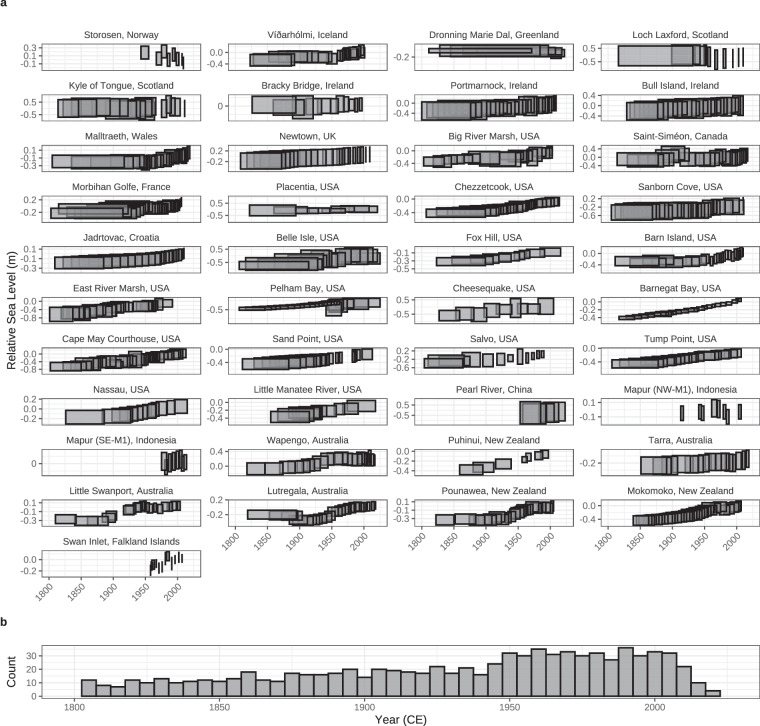
(**a**) Relative sea-level reconstructions from the P-1800SLD^45^ with all continuous sea-level index point ages calibrated to IntCal20 or SHCal20 and all age-depth models run in rplum/rbacon. Sites ordered by descending latitude (North–South). Vertical estimates of relative sea level remain as originally published. Note the variable y axes scaling. Errors given to 2σ. (**b**) Histogram of updated mean ages for Sea-Level Index Points. Data shown are limited to between 1800–2025 CE with 5-year bins.

In P-1800SLD^45^, the majority of SLIPs fall within the period ~1950–2010 CE (Fig. 4). Prior to ~1900 CE, there is a pronounced Northern Hemisphere bias, with ~75–80% of SLIPs originating from sites located in the North Atlantic. While SLIPs from the Indo-Pacific are present pre-1900 CE, their representation remains limited until a marked increase after 1950 CE, after which they typically account for ~30–50%. The number of Indo-Pacific SLIPs post-2010 CE reflects, in part, recent intensified efforts to investigate and document sea-level changes in these under-represented regions. While SLIPs across the database generally exhibit a trend of rising RSL from 1800–2025 CE, the signal is characterised by significant local and regional variability. Such variability arises from linear (e.g., postglacial rebound) and non-linear processes including local oceanographic dynamics and the effects of gravitational, rotational and deformational adjustments following ice sheet and land-based glacier melt. The database does not apply corrections for any aspect of glacio-isostatic adjustment or vertical land motion, allowing users to explore the full sea-level budget.

## Methods

A review of published high-resolution proxy-based Common Era sea-level reconstructions was conducted to develop the chronologically consistent database. In summary, the database was constructed as follows:

### Site identification

We conducted a literature review to identify and synthesise relevant high-resolution, post-1800 CE sea-level reconstructions for inclusion in the database. Key words were identified using titles of papers from the most recent Common Era database (W21^2^), ensuring comprehensive coverage of terminology commonly associated with high-resolution sea-level reconstructions and the wider discipline. We created a Boolean search query and conducted searches using Google Scholar and Web of Science. The resulting search term was: “sea level” OR “relative sea level” AND (proxy OR reconstruction OR “salt marsh” OR “saltmarsh” OR “foraminifera” OR “diatoms” OR “microatoll” OR “mangrove”) AND (“Common Era” OR “last 2000 years” OR “late Holocene” OR “19^th^ century” OR “20^th^ century” OR “last 200 years”). Articles were sorted by relevance, and a manual search was then conducted to identify papers of relevance from the search until the relevance of the papers ceased. Additionally, we used a ‘snowballing’ approach, following citations within relevant papers to find further studies for inclusion.

Here, we define “relevant high-resolution sea-level reconstructions” as those with at least four index points in the reference period 1950 CE–2000 CE and those which have a mean SLIP age uncertainty of ≤50 years and a mean timestep of ≤25 years. Requiring at least four SLIPs in the period 1950 to 2000 CE, emerges from our objective of harmonising SLIP reconstructions with tide-gauge observations using a common reference time period rather than a vertical datum. Our requirement that reconstructions have a mean SLIP age uncertainty of ≤50 years and a mean timestep of 25 years reflects the resolution needed to support research on post-industrial sea-level acceleration i.e., the “timing of emergence” and deviation from the Holocene background rate^15^, and timescales of ocean-climate interaction. Age uncertainty links to the sample age, while mean timestep links to SLIP density and time between SLIPs. These SLIP factors are indirectly connected, but both will affect subsequent analyses by obscuring or clarifying the temporal evolution of RSL as recorded by SLIPs. Many post-1800 CE SLIPs are dated using short-lived isotopes (e.g., ¹³⁷Cs, ^210^Pb), pollen markers (e.g., *Ambrosia, Pinus*) or historical chronohorizons (e.g., tephra and pollutants) which routinely achieve < 50-year uncertainties. In total, we identified 49 relevant sites from the literature (41 Northern in the Northern Hemisphere, 8 in the Southern).

Having identified the prospective sites, we sourced the original, publicly available data including data for age-depth modelling, or we requested data from the corresponding author if they were not available in the original publication. The following sites were not included as all raw data needed to complete an age-depth model update were not publicly available and could not be sourced (see Chronology updates section): Swan and Snipe Key, USA^26^, Les Sillons and Bassin, Canada^52^, Plentzia Estuary^53^, Muskiz Estuary^54^ and Urdaibai Estuary^55^, Spain and the Huvadhoo atoll, Maldives^56,57^.

### Chronology updates

Periodic updates to the radiocarbon calibration curve^40,41^, driven by the availability of new data and advancements in statistical modelling, necessitate recalibration of radiocarbon ages of reconstructions within the database. These updates enhance the accuracy and precision of subsequent relative sea-level reconstructions, thereby increasing confidence when comparing them to observational data or calculating regional sea-level budgets. We first downloaded the original data and tabulated into a consistent format via the source publication or the corresponding author. We then identified sites for which recalibration and/or a new age-depth model was needed and constructed an input file for the age-depth model using ^14^C and ^210^Pb (if available). For sites where chronostratigraphic data were available, we added calendar ages to the input file at the mid-point of the horizon’s depth range.

In 2020, calibration curves were re-designed using Bayesian splines and Markov-Chain Monte-Carlo (MCMC) sampling (which replaced the previous random walk method)^58^. Thus, we recalibrate sites originally calibrated prior to 2020, and sites not originally modelled using rplum or rbacon (depending on the availability of ^210^Pb radionuclide data) for model consistency. Whilst there is no integrated depth uncertainty function in rplum or rbacon, we prefer them given the ability to model raw ^210^Pb data in a Bayesian framework, which is not currently possible in other models (these must input modelled ^210^Pb ages as calendar ages).

We produced new age-depth models for 71% (n = 29) of the preliminary database. Exceptions included where, 1. the original study used rplum or rbacon and the IntCal20/SHCal20^40,41^ calibration curves (24%; n = 10), or 2. no radiocarbon data or ^210^Pb were used in the construction of the reconstruction (5%; n = 2). Where necessary, appropriate post-bomb calibration curves^42^ were applied. If chronostratigraphic markers with calendar dates were available from original age-depth models, they were incorporated into the models, assuming a normal distribution. Where chronostratigraphic markers have an associated depth uncertainty, we use the mid-point of the range (full depth ranges can be viewed in the chronostratigraphic marker tab).

All input data (i.e., the raw ^14^C data, ^210^Pb and chronostratigraphic markers) to the age-depth models remains the same as that originally published, including ages earlier than 1800 CE (see for example Tab 3 – Raw_^14^C_Data). The exception was if an uncertainty was not provided for ^137^Cs bomb spike date (~1963 CE) a ± 2-year uncertainty was added. Whilst we calculate all posterior ages, we limit the processed output to 1800–2025 CE to support investigations of decadal to multidecadal sea-level changes and maximise spatial coverage (the distribution of high-resolution sites skews toward the Northern Hemisphere prior to 1800 CE). This period ensures sufficient chronological resolution and minimal age uncertainty, as large temporal hiatuses and age uncertainties in older data (i.e., pre-1800) can obscure shorter-term sea-level variability. All available raw age data are provided in Tabs 3–5, allowing users to explore the full dataset if desired.

We ran the models with default priors and an accumulation rate suggested by rplum/rbacon. At the Loch Laxford^59^ site, two sediment cores–LA3 and LA6–were originally collected. We developed new age-depth models for both cores. However, for core LA6, the modelled ^210^Pb chronology could not be reconciled with the measured ^210^Pb activity profile (likely as a result of the core not reaching background). Consequently, LA6 was excluded from the final dataset due to the unreliability of its age–depth reconstruction, but LA3 was retained.

Finally, we visually inspected the modelled output to ensure the age–depth model was appropriate, verifying that the modelled ^210^Pb estimates aligned with the measured values and that the log-objective plot (reflecting the MCMC process) showed no structure. Systematic oscillations or pronounced fluctuations in the log-objective plot can indicate convergence issues or model misfit, in which case the model should be re-run, or the number of MCMC iterations should be increased. For further information, see the reference manual documentation associated with the rplum/bacon packages available on CRAN. If the model was appropriate, we extracted the posterior ages for each depth with a palaeomarsh surface elevation (PMSE) estimate and associated RSL data (note rplum/rbacon solve calibration and age-depth model within the same function) with a normal age approximation (i.e., mean and 2 standard deviations).

### Quality control: Comparison of original and new age-depth models and sea-level reconstructions

**Fig. 5:**
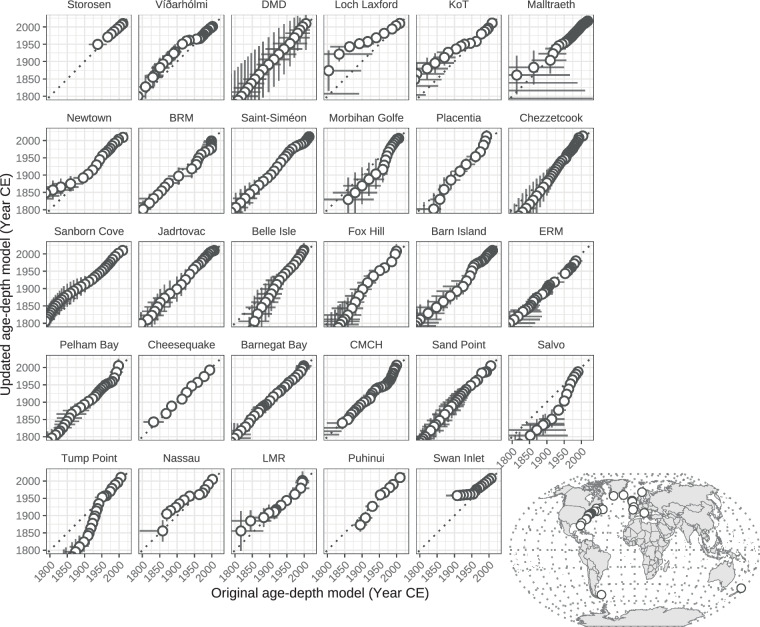
Comparison of original and updated age-depth models. Sites ordered by descending latitude (North–South). Errors given to 2σ. DMD = Dronning Marie Dal, BRM = Big River Marsh, ERM = East River Marsh, CMCH = Cape May Courthouse, LMR = Little Manatee River.

We next compare original and new age-depth models to identify sites that deviate substantially from a 1:1 relationship (Fig. 5) and review these on a case-by-case basis. In most cases, our outputted normal age error approximation closely resembles the ages originally published, but with six notable exceptions: Loch Laxford and Kyle of Tongue, Scotland (UK)^59^, Newtown Marsh, Isle of Wight (UK)^60^), Swan Inlet, Falkland Islands^25,61^, and Tump Point^62,63^ and Salvo, USA^64^.

For Loch Laxford, Kyle of Tongue, Newtown and Salvo, the divergence in ages for these sites may be due to the use of linear regression to model ^210^Pb in the original publications, while the new analysis employs a Bayesian method. The rplum model assumes a constant rate of supply of ^210^Pb, but separates the age-depth modelling process from the ^210^Pb decay equation by using a self-adjusting MCMC algorithm^65^. Additionally, the updated age-depth models account for background levels not being reached and estimate background, which may have led to discrepancies in the age-depth estimates. Compared to the traditional Constant Rate of Supply (CRS) or Constant Initial Concentration (CIC) models, rplum offers improved accuracy by incorporating priors, allowing for variable sedimentation rates and integrating multiple dating approaches. Accordingly, we retain these revised models in the database under the assumption that the updated chronologies are more accurate due to methodological improvements since the original published ages.

While the updated ages at Swan Inlet are estimated younger than the original SHCal13 estimates, it is clear from Table 6.7 and the accompanying discussion in the original thesis (Newton^61^) that high-precision bomb-spike ^14^C dates drive the chronology in this section of the model. Specifically, 13 of 19 ^14^C determinations in the top 90 cm of the core fall within the bomb-spike era. In combination with the ^137^Cs chronostratigraphic marker dated to 1964–1965 CE at 6–8 cm, all updated SLIP ages in the top 15 cm of the core therefore fall in the latter half of the 20^th^ century with very small age uncertainty. Finally, there is a discrepancy between the Tump Point core original and updated age-depth model, with deviations in age estimates before ~1925 CE. This discrepancy is likely driven by the limited chronological constraint in this part of the core and model specificity^30,39^.

SLIP age estimates between the original and updated models are remarkably consistent, with an overall average difference of 2 years in mean age. Barn Island, USA exhibits the smallest discrepancy between mean age estimates (average less than a year), whereas Tump Point, USA has the largest (average 38 years). In general, ages diverge more significantly the further back in time, particularly prior to 1900 CE. Chronostratigraphic markers – such as introduction of exotic pollen, the use and subsequent phase-out of leaded petrol, the onset and peak of atmospheric nuclear weapons testing and other well-documented pollution events – act as tie points between models. These events provide critical prior information that clearly suppresses model-specific behaviour (which can affect, for example, which part of a ^14^C age distribution a model samples from, which can result in disparate age estimates for the same depth from the same ^14^C date). As such, these markers significantly reduce age variability and enforce consistency between models. We believe it is the presence of these markers that has led to the convergence of age estimates for SLIPs dated from the 19^th^ to 21^st^ centuries.

Although generally small, model-specific discrepancies in SLIP age, particularly those from the early-mid 19^th^ century, underscore the importance of consistency in both the age-depth model and the calibration curve for inter-site comparisons. This is particularly critical when attempting to resolve sub-decadal sea-level variations, as differences in age uncertainty estimates—driven by model specifications or calibration curve discrepancy—could influence interpretations. These differences may affect estimates of rates, magnitudes and the timing of sea-level acceleration in regional and global sea-level reconstructions. Users may therefore wish to repeat this exercise using Bchron for comparison using the raw data provided.

Finally, challenges arise when databases include both normal and non-normal error approximations, as these represent fundamentally different sources of uncertainty, which could lead to inconsistent interpretations of SLIPs. Although the approach may be considered a simplification, we output normal posterior age uncertainties as this approximation is required for errors-in-variables regression or Gaussian process regression modelling—methods frequently utilised in the subsequent stages of analysis. However, as shown in Fig. 5, this approximation does not differ substantially from the original uncertainty estimates, supporting the updated SLIPs’ suitability for further modelling.

## Data Records

The P-1800SLD database^45^ (10.6084/m9.figshare.30112906) is structured into seven tabs, each serving a specific purpose to assist users in understanding, analysing, and reprocessing SLIP data. The first tab, READ_ME, provides guidance and instructions that users should consult before downloading or working with the dataset. It outlines the structure of the database and offers context for how the data should be interpreted and used. The Summary tab contains metadata for each site included in the database. This includes details such as the site name, region, latitude and longitude, SLIP classification, and a summary of the age data available for each model. It also indicates whether the age model was updated from its originally published version, specifies which modelling approach was used (either rplum or rbacon), and includes any additional comments that may be of use to the user. Three tabs contain the raw input data used in the modelling process. The Raw_^14^C_Data tab includes original radiocarbon data (or where a bomb-spike date has been converted to a radiocarbon date, the original data expressed as ‘fraction modern’ are given), while the Raw_^210^Pb_Data tab contains ^210^Pb data and bulk density data for sites where this method was applied. The Raw_Chronostratigraphic_Data contains information on chronostratigraphic markers that were used to constrain the age-depth models, as well as any depth uncertainties (if applicable). These raw data tabs are useful for users who wish to re-run models using updated calibration curves or alternative modelling frameworks such as Bchron or OxCal. The Processed_Age_Data + RSL tab presents the final outputs of the modelling process. It includes original RSL estimates alongside posterior modelled ages. This tab also provides the original PMSE depths should a user wish to undertake an update. Finally, the References tab lists all original sources from which data were extracted to construct the database.

## Technical Validation

### Comparison with tide gauges

Tide-gauge (TG) records provide an independent dataset to assess the ability of high-resolution reconstructions (where the two sites are located in close proximity i.e., co-located) to represent RSL, and associated trends and variability. Factors influencing the agreement between datasets include site locations (open coast vs estuarine environments) and tidal regime (amplified or damped based on coastal setting). Therefore, the intercomparison presented here provides an indication of coherent sea-level behaviour (i.e., do both datasets capture the same signal?). Temporal uncertainty for TG records, compared to modelled and measured SLIPs, is negligible, while the time-mean sea level is considered highly accurate (low uncertainty) owing to the high-frequency sampling (hourly or 6-minute) and short duration of tidal cycles (hours-days) and hydro-meteorological effects (hours), which are averaged out over the monthly and annual timescales that are published.

We use annual TG data from the Permanent Service For Mean Sea Level (PSMSL)^66^ and identify stations that are <100 km from proxy reconstruction sites with long and continuous records (>60 years, >70% complete) that end after 2010. If multiple TGs fulfilled these criteria, we selected the gauge with the smallest inter-site distance to the proxy location, supplemented by visual inspection of maps to verify that TG-proxy paired sites are located along the same coastal transect and exposed to similar ocean dynamics. No SLIP sites fulfil these criteria from the Indo-Pacific region because their nearest TGs are either too short, lack completeness, or are too distant. The intercomparison here does not invalidate the Indo-Pacific sites, but retaining these criteria is necessary for an objective validation. As such, all SLIPs in the intercomparison are modelled SLIPs.

**Fig. 6:**
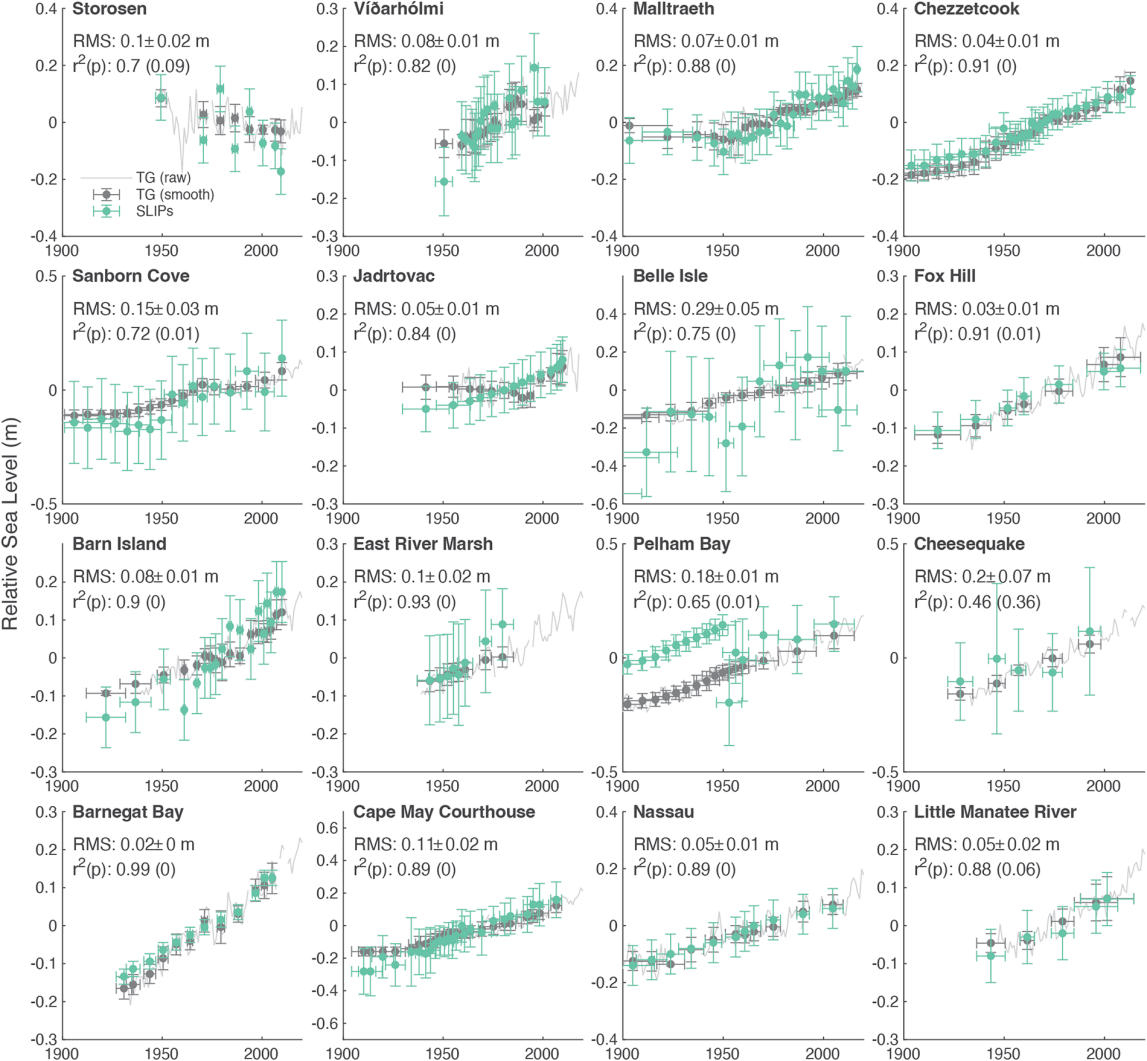
SLIP validation against tide gauges for criteria 1 (water levels relative to 1950–2000 average). Revised SLIPs from this study (green) are plotted with the annual tide gauge record (light grey) and time averaged over each SLIP age range (dark grey, TG (smooth)). RMS misfit and correlation (r^2^, p-value) are averages of the Monte-Carlo sampling. Note variable y axis.

Annual tide-gauge RSL measurements are not directly comparable to SLIP-derived RSL estimates for three reasons, which we describe in turn and outline the post-processing steps to address them. The adjustments described below produce a new type of metric – a tide-gauge equivalent SLIP (hereafter TG-SLIP), derived from the tide-gauge record but constructed to be directly comparable with the corresponding SLIP (Fig. 6).

First, SLIPs and TGs are usually measured relative to different vertical datums, leading to offsets in elevation. As previously mentioned, we address this by recalculating RSL for both data relative to the same reference period (1950–2000 CE) by subtracting the time mean of data at each site in that period from all data at that site.

Second, TGs and SLIPs have different age uncertainties that must be reconciled to allow direct comparison. We construct TG-SLIPs by averaging local annual tide gauge RSL values over the age range of each SLIP. For example, for a SLIP dated 1850 ± 20 CE, the TG-SLIP RSL is calculated by averaging the annual tide-gauge record from 1830 to 1870 CE. Likewise, the RSL uncertainty of the TG-SLIP is calculated as the standard deviation of annual RSL values over the same period.

Third, the RSL uncertainties of TGs and SLIPs do not include the same sources of error. The TG-SLIP RSL uncertainty reflects the interannual variability over the SLIP age range, whereas the SLIP RSL uncertainty includes the indicative range (the ecological/physical relationship to a specific water level, often linked to tidal range), elevation errors, core shortening effects (e.g., via compaction), and sample thickness^67^. To account for this difference, we adjust the SLIP RSL uncertainty to incorporate the interannual variability derived from the tide-gauge record (${\sigma }_{{SLIP}* }^{2}={\sigma }_{{SLIP}}^{2}+{\sigma }_{{TG}-{SLIP}}^{2}$). This leads to a centimetre-scale increase in SLIP RSL uncertainty and simplifies the intercomparison between TG-SLIP and SLIP data.

**Fig. 7:**
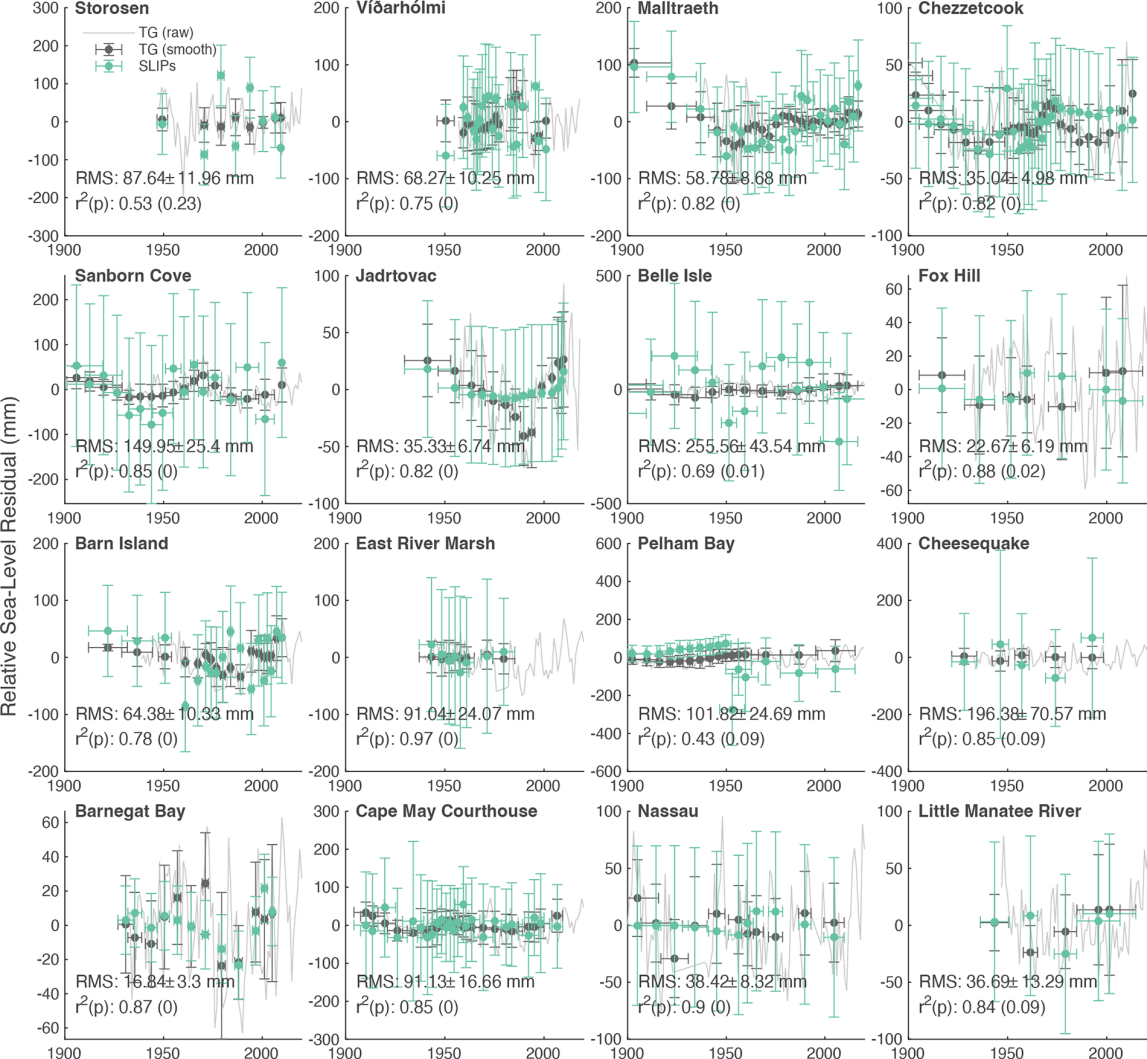
Comparison of RSL residuals from SLIP sites and tide gauges. Residuals are calculated by linearly detrending each dataset respectively. Revised SLIPs from this study (green) are plotted with the annual tide gauge record (light grey) and time averaged over each SLIP age range (dark grey, TG (smooth)). RMS misfit and correlation (r^2^, p-value) are averages of the Monte-Carlo sampling. Note variable y axis.

**Fig. 8:**
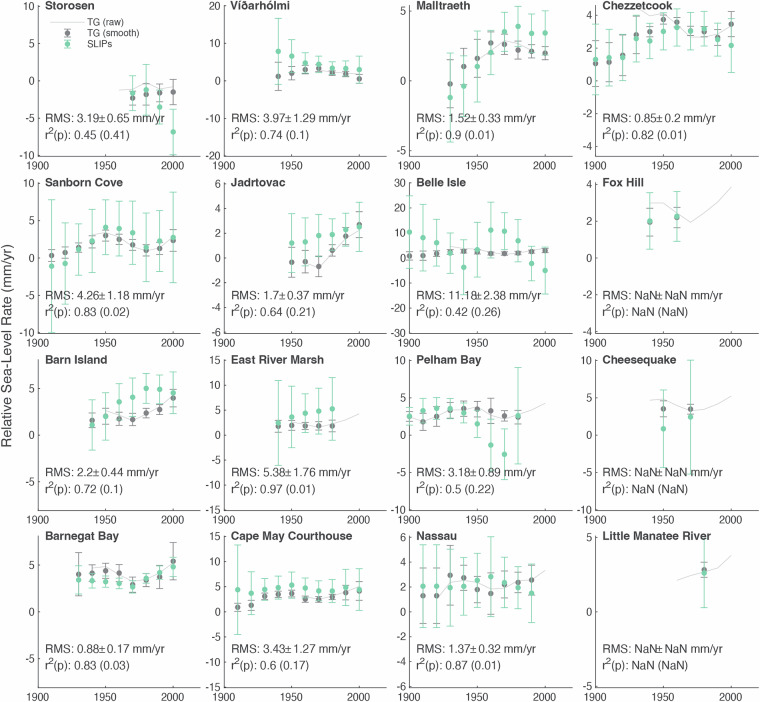
SLIP validation against tide gauges for criteria 2 (RSL rates at decadal timestep for 50-year windows). RSL rates of revised SLIPs from this study (green) are plotted with the raw tide gauge RSL rate (light grey) and the SLIP age-range, time averaged tide gauge RSL rate (dark grey, TG (smooth). RMS misfit and correlation (r^2^, p-value) are averages of the Monte-Carlo sampling. Note variable y axis.

We set out three tests to validate the SLIPs with TG-SLIP data. Standard statistical measures (correlation, root-mean-square error (RMSE), and standard deviation) are applied for (1) SLIP and TG-SLIP water levels (including detrended residuals) (Figs. 6, 7), and (2) SLIP and TG-SLIP RSL rates (Fig. 8). For test 2, we use linear regression accounting for both vertical and temporal uncertainties^68^ to estimate RSL rates from each data type every 10 years using a 50-year sliding window. In all tests, the statistical quantities are solved using a Monte-Carlo framework (n = 10,000) to account for uncertainties in time/elevation. Each test allows us to assess the accuracy and utility of the SLIP data to represent water level and trends against the baseline of instrumental tide-gauge data.

Sixteen locations within the SLIP database satisfy the criteria for TG intercomparison (Table 2). These are all located in the western North Atlantic owing to the presence of long, complete tide gauges with good spatial coverage. The average SLIP to TG distance was 33 km, varying from 6 to 72 km (Fox Hill and Viðarhólmi respectively), although, it is not apparent that increased misfit/decreased correlation occurred with distance. For test 1 (water levels), SLIPs and TG-SLIPs show very good agreement over the 20^th^ century, with average correlations of 0.82 and average RMSE of 0.10 m (Fig. 6). Similarly, detrended water levels also show average correlations of 0.79 (Fig. 7).

**Table 2:** Proxy site and PSMSL tide-gauge pairings used for the validation exercise including instrument time span and inter-site distance.

Site	PSMSL TG (ID)	TG range (years)	Inter-site distance (km)
Storosen	Narvik (45)	1948–2025	52.3
Víðarhólmi	Reykjavik (638)	1957–2018	72.3
Malltraeth	Holyhead (5)	1938–2024	22.7
Sanborn Cove	Eastport (332)	1930–2021	41.3
Jadrtovac	Split – Gradska Luka (352)	1955–2018	43.6
Belle Isle	Boston (235)	1921–2021	6.4
Chezzetcook	Halifax (96)	1895–2012	26.9
Fox Hill	Newport (351)	1930–2025	5.9
Barn Island	New London (429)	1938–2025	18.9
East River Marsh	New London (429)	1938–2025	48.0
Pelham Bay	New York – The Battery (12)	1856–2021	25.6
Cheesequake	Sandy Hook (366)	1932–2025	22.4
Barnegat Bay	Sandy Hook (366)	1932–2025	48.9
Cape May Courthouse	Lewes (Breakwater Harbor) (224)	1919–2025	43.1
Nassau	Fernandina Beach (112)	1898–2021	21.4
Little Manatee River	St Petersburg (520)	1947–2021	18.5

A key result of this analysis is that most sites track both time averaged mean sea level and interannual variability, though their uncertainties vary between that of the TG-SLIP up to an order of magnitude greater (e.g., compare the size of the error bars for Cheesequake). In addition, some SLIP sites show greater variability though time than that calculated by the TG despite SLIP vertical uncertainties overlapping them (e.g., Belle Isle). In some locations, and despite similar uncertainties and trends, there are apparent offsets that may indicate localised sea level differences or SLIP elevation measurement inconsistencies (e.g., Pelham Bay). These results provide some insight into the interpretability of high-resolution sea-level reconstructions. Where possible, an intercomparison with a nearby tide gauge allows one to assess whether SLIPs capture mean sea level variability per se, or if they impose variability that might be misinterpreted. Likewise, they offer the chance to identify local effects (e.g., offsets/trends). Finally, a coupled analysis of water levels and trends allows for full utility of the SLIPs. While they may not be perfect geological tide gauges, they provide a reasonable approximation at this timescale and serve as valuable indicators for extending sea-level records prior to the instrumental era.

The P-1800SLD^45^ serves as a centralised, updated database of post-1800 CE sea-level data, enabling investigation of post-industrial spatio-temporal sea-level change, analysis of regional and global sea-level budgets, and supplementation of tide-gauge records to extend coverage into the 19th century. The inclusion of raw chronological data for all sites ensures the database remains adaptable and updatable in response to: (1) the publication of new high-resolution sites that meet the outlined criteria; (2) improvements to calibration curves or age-depth modelling techniques; and (3) the application of alternative age-depth modelling approaches.

## Data Availability

The datasets supporting this study are openly available as follows: • The P-1800SLD dataset is available via Figshare at: 10.6084/m9.figshare.30112906. • The tide-gauge data used in this study can be accessed through the Permanent Service for Mean Sea Level (PSMSL) at https://psmsl.org/data/. All data are publicly accessible and can be reused under the terms provided by the respective repositories.

## Code availability

All software and pipelines were executed according to the methods section. No custom code was generated for this study.

## References

[1] Shennan, I., Bradley, S. L. & Edwards, R. Relative sea-level changes and crustal movements in Britain and Ireland since the Last Glacial Maximum. *Quat. Sci. Rev.* **188**, 143–159 (2018).

[2] Walker, J. S. *et al*. Common Era sea-level budgets along the U.S. Atlantic coast. *Nat. Commun.* **12**, 1841 (2021). 33758184 10.1038/s41467-021-22079-2 PMC7988146

[3] Horton, B. P. *et al*. Predicting marsh vulnerability to sea-level rise using Holocene relative sea-level data. *Nat. Commun.* **9**, 2687 (2018). 30002365 10.1038/s41467-018-05080-0 PMC6043595

[4] Barnett, R. L. *et al*. Nonlinear landscape and cultural response to sea-level rise. *Sci. Adv.* **6**, eabb6376 (2020). 33148641 10.1126/sciadv.abb6376 PMC7673675

[5] Rovere, A. *et al*. The World Atlas of Last Interglacial Shorelines (version 1.0). *Earth Syst. Sci. Data* **15**, 1–23 (2023).

[6] Hibbert, F. D., Williams, F. H., Fallon, S. J. & Rohling, E. J. A database of biological and geomorphological sea-level markers from the Last Glacial Maximum to present. *Sci. Data* **5**, 180088 (2018). 29809175 10.1038/sdata.2018.88 PMC5972710

[7] Khan, N. S. *et al*. Inception of a global atlas of sea levels since the Last Glacial Maximum. *Quat. Sci. Rev.* **220**, 359–371 (2019).

[8] Engelhart, S. E. & Horton, B. P. Holocene sea level database for the Atlantic coast of the United States. *Quat. Sci. Rev.* **54**, 12–25 (2012).

[9] Kopp, R. E. *et al*. Temperature-driven global sea-level variability in the Common Era. *Proc. Natl. Acad. Sci. USA.* **113**, E1434–41 (2016). 26903659 10.1073/pnas.1517056113 PMC4801270

[10] Kemp, A. C. *et al*. Relative sea-level change in Newfoundland, Canada during the past ∼3000 years. *Quat. Sci. Rev.* **201**, 89–110 (2018).

[11] Kemp, A. C., Wright, A. J. & Cahill, N. Enough is Enough, or More is More? Testing the Influence of Foraminiferal Count Size on Reconstructions of Paleo-Marsh Elevation. *J. Foraminifer. Res.* **50**, 266–278 (2020).

[12] Kemp, A. C., Shaw, T. A. & Piecuch, C. G. The importance of non-tidal water-level variability for reconstructing Holocene relative sea level. *Quat. Sci. Rev.* **290**, 107637 (2022).

[13] Woodroffe, S. A. *et al*. Missing sea level rise in southeastern Greenland during and since the Little Ice Age. *Clim. Past* **19**, 1585–1606 (2023).

[14] Kemp, A. C., Dutton, A. & Raymo, M. E. Paleo Constraints on Future Sea-Level Rise. *Curr. Clim. Change Rep.* **1**, 205–215 (2015).

[15] Walker, J. S., Kopp, R. E., Little, C. M. & Horton, B. P. Timing of emergence of modern rates of sea-level rise by 1863. *Nat. Commun.* **13**, 966 (2022). 35181652 10.1038/s41467-022-28564-6 PMC8857177

[16] Hijma, M. P. *et al*. A protocol for a geological sea-level database. in *Handbook of Sea-Level Research* 536–553 (John Wiley & Sons, Ltd, 2015).

[17] Shennan, I. Handbook of sea-level research. in *Handbook of Sea-Level Research* 3–25 (John Wiley & Sons, Ltd, 2015).

[18] Vacchi, M. *et al*. Multiproxy assessment of Holocene relative sea-level changes in the western Mediterranean: Sea-level variability and improvements in the definition of the isostatic signal. *Earth-Sci. Rev.* **155**, 172–197 (2016).

[19] Walker, J. S. & Cahill, N. Influence of Foraminifera Count Size and Rare Species on Transfer Function Results Used in Sea-Level Reconstructions. *J. Foraminifer. Res.* **54**, 107–116 (2024).

[20] Kemp, A. C. & Telford, R. J. Transfer functions. in *Handbook of Sea-Level Research* (John Wiley & Sons, 2015).

[21] Brain, M. J. *et al*. Exploring mechanisms of compaction in salt-marsh sediments using Common Era relative sea-level reconstructions. *Quat. Sci. Rev.* **167**, 96–111 (2017).

[22] Parnell, A. C. & Gehrels, W. R. Using chronological models in late Holocene sea-level reconstructions from saltmarsh sediments. in *Handbook of Sea-Level Research* 500–513 (John Wiley & Sons, Ltd, 2015).

[23] Barnett, R. L., Gehrels, W. R., Charman, D. J., Saher, M. H. & Marshall, W. A. Late Holocene sea-level change in Arctic Norway. *Quat. Sci. Rev.* **107**, 214–230 (2015).

[24] Williams, S. L. *et al*. Relative sea-level changes in southeastern Australia during the 19th and 20th centuries. *J. Quat. Sci.* **38**, 1184–1201 (2023).

[25] Frederikse, T. *et al*. Constraining 20th-Century Sea-Level Rise in the South Atlantic Ocean. *J. Geophys. Res. Oceans* **126**, e2020JC016970 (2021).

[26] Khan, N. S. *et al*. Relative sea-level change in South Florida during the past ~5000 years. *Glob. Planet. Change* **216**, 103902 (2022).

[27] Majewski, J. M. *et al*. Extending Instrumental Sea-Level Records Using Coral Microatolls, an Example From Southeast Asia. *Geophys. Res. Lett.* **49**, e2021GL095710 (2022).

[28] Parnell, A. C., Buck, C. E. & Doan, T. K. A review of statistical chronology models for high-resolution, proxy-based Holocene palaeoenvironmental reconstruction. *Quat. Sci. Rev.* **30**, 2948–2960 (2011).

[29] Cahill, N., Kemp, A. C., Horton, B. P. & Parnell, A. C. A Bayesian hierarchical model for reconstructing relative sea level: from raw data to rates of change. *Clim. Past* **12**, 525–542 (2016).

[30] Wright, A. J. *et al*. Reconstructing the accumulation history of a saltmarsh sediment core: Which age-depth model is best? *Quat. Geochronol.* **39**, 35–67 (2017).

[31] Stearns, R. B. *et al*. Within-region replication of late Holocene relative sea-level change: An example from southern New England, United States. *Quat. Sci. Rev.* **300**, 107868 (2023).

[32] Roseby, Z. A. *et al*. Two Centuries of Relative Sea-Level Rise in Dublin, Ireland, Reconstructed by Geological Tide Gauge. *Open Quat.* **9**, 3 (2023).

[33] Blaauw, M., Christen, J. A., Bennett, K. D. & Reimer, P. J. Double the dates and go for Bayes — Impacts of model choice, dating density and quality on chronologies. *Quat. Sci. Rev.* **188**, 58–66 (2018).

[34] Aquino-López, M. A., Ruiz-Fernández, A. C., Blaauw, M. & Sanchez-Cabeza, J.-A. Comparing classical and Bayesian 210Pb dating models in human-impacted aquatic environments. *Quat. Geochronol.* **60**, 101106 (2020).

[35] Blaauw, M. & Christen, J. A. Flexible paleoclimate age-depth models using an autoregressive gamma process. *Bayesian Anal.* **6**, 457–474 (2011).

[36] Haslett, J. & Parnell, A. A Simple Monotone Process with Application to Radiocarbon-Dated Depth Chronologies. *J. R. Stat. Soc. Ser. C Appl. Stat.* **57**, 399–418 (2008).

[37] Ramsey, C. B. Radiocarbon Calibration and Analysis of Stratigraphy: The OxCal Program. *Radiocarbon* **37**, 425–430 (1995).

[38] Parnell, A. C., Haslett, J., Allen, J. R. M., Buck, C. E. & Huntley, B. A flexible approach to assessing synchroneity of past events using Bayesian reconstructions of sedimentation history. *Quat. Sci. Rev.* **27**, 1872–1885 (2008).

[39] Trachsel, M. & Telford, R. J. All age–depth models are wrong, but are getting better. *The Holocene* **27**, 860–869 (2017).

[40] Hogg, A. G. *et al*. SHCal20 Southern Hemisphere calibration, 0–55,000 years cal BP. *Radiocarbon* **62**, 759–778 (2020).

[41] Reimer, P. J. *et al*. The IntCal20 Northern Hemisphere Radiocarbon Age Calibration Curve (0–55 cal kBP). *Radiocarbon* **62**, 725–757 (2020).

[42] Hua, Q. *et al*. Atmospheric radiocarbon for the period 1950–2019. *Radiocarbon* **64**, 723–745 (2022).

[43] Barker, M. *et al*. Introducing the FAIR Principles for research software. *Sci. Data* **9**, 622 (2022). 36241754 10.1038/s41597-022-01710-x PMC9562067

[44] HOLSEA Datahub. https://holsea-datahub-alpha.vercel.app/ (2026).

[45] Williams, S. L., Jackson, L. P., Garrett, E., Hibbert, F. D. & Gehrels, W. R. A global chronologically standardised database of high-resolution proxy sea-level reconstructions since 1800 CE. *Figshare*10.6084/m9.figshare.30112906.v1 (2026). 10.1038/s41597-026-07232-0 PMC13287787 42020484

[46] Carton, J. A., Chepurin, G. A. & Chen, L. SODA3: A New Ocean Climate Reanalysis. *J. Clim.* **31**, 6967–6983 (2018).

[47] Griffies, S. M. *et al*. OMIP contribution to CMIP6: experimental and diagnostic protocol for the physical component of the Ocean Model Intercomparison Project. *Geosci. Model Dev.* **9**, 3231–3296 (2016).

[48] Jungclaus, J. H. *et al*. The PMIP4 contribution to CMIP6 – Part 3: The last millennium, scientific objective, and experimental design for the PMIP4 *past 1000* simulations. *Geosci. Model Dev.* **10**, 4005–4033 (2017).

[49] Wright, A. J., Edwards, R. J. & van de Plassche, O. Reassessing transfer-function performance in sea-level reconstruction based on benthic salt-marsh foraminifera from the Atlantic coast of NE North America. *Mar. Micropaleontol.* **81**, 43–62 (2011).

[50] Williams, S., Garrett, E., Moss, P., Bartlett, R. & Gehrels, R. Development of a Regional Training Set of Contemporary Salt-Marsh Foraminifera for Late Holocene Sea-Level Reconstructions in southeastern Australia. *Open Quat*. **7**, (2021).

[51] Kemp, A. C., Cahill, N., Engelhart, S. E., Hawkes, A. D. & Wang, K. Revising Estimates of Spatially Variable Subsidence during the A.D. 1700 Cascadia Earthquake Using a Bayesian Foraminiferal Transfer Function. *Bull. Seismol. Soc. Am.* **108**, 654–673 (2018).

[52] Barnett, R. L., Bernatchez, P., Garneau, M. & Juneau, M.-N. Reconstructing late Holocene relative sea-level changes at the Magdalen Islands (Gulf of St. Lawrence, Canada) using multi-proxy analyses. *J. Quat. Sci.* **32**, 380–395 (2017).

[53] Leorri, E., Horton, B. P. & Cearreta, A. Development of a foraminifera-based transfer function in the Basque marshes, N. Spain: implications for sea-level studies in the Bay of Biscay. *Mar. Geol.* **251**, 60–74 (2008).

[54] Leorri, E. & Cearreta, A. Recent sea-level changes in the southern Bay of Biscay: transfer function reconstructions from salt-marshes compared with instrumental data. *Sci. Mar.* **73**, 287–296 (2009).

[55] García-Artola, A., Cearreta, A., Leorri, E., Irabien, M. J. & Blake, W. H. Las marismas costeras como archivos geológicos de las variaciones recientes en el nivel marino. *Geogaceta* **47**, 109–112 (2009).

[56] Kench, P. S. *et al*. Climate-forced sea-level lowstands in the Indian Ocean during the last two millennia. *Nat. Geosci.* **13**, 61–64 (2020).

[57] Kench, P. S. *et al*. Coral growth records 20th Century sea-level acceleration and climatic variability in the Indian Ocean. *Nat. Commun.* **16**, 5872 (2025). 40592882 10.1038/s41467-025-60972-2 PMC12217869

[58] Reimer, P. J. Evolution of radiocarbon calibration. *Radiocarbon* **64**, 523–539 (2022).

[59] Barlow, N. L. *et al*. Salt-marsh reconstructions of relative sea-level change in the North Atlantic during the last 2000 years. *Quat. Sci. Rev.* **99**, 1–16 (2014).

[60] Long, A. J. *et al*. Contrasting records of sea-level change in the eastern and western North Atlantic during the last 300 years. *Earth Planet. Sci. Lett.* **388**, 110–122 (2014).

[61] Newton, T. L. Holocene sea-level changes in the Falkland Islands: new insights into accelerated sea-level rise in the 20th Century. (University of Plymouth, 2017).

[62] Kemp, A. C. *et al*. Climate related sea-level variations over the past two millennia. *Proc. Natl. Acad. Sci.* **108**, 11017–11022 (2011). 21690367 10.1073/pnas.1015619108 PMC3131350

[63] Kemp, A. C. *et al*. Extended late Holocene relative sea-level histories for North Carolina, USA. *Quat. Sci. Rev.* **160**, 13–30 (2017).

[64] Kemp, A. C. *et al*. The relative utility of foraminifera and diatoms for reconstructing late Holocene sea-level change in North Carolina, USA. *Quat. Res.* **71**, 9–21 (2009).

[65] Sim, T. G. *et al*. Ecology of peatland testate amoebae in Svalbard and the development of transfer functions for reconstructing past water-table depth and pH. *Ecol. Indic.* **131**, 108122 (2021).

[66] Hogarth, P. Preliminary analysis of acceleration of sea level rise through the twentieth century using extended tide gauge data sets (August 2014). *J. Geophys. Res. Oceans* **119**, 7645–7659 (2014).

[67] Khan, N. S. Databases of sea-level change. in *Encyclopedia of Quaternary Science* 174–192 (Elsevier, 2025).

[68] York, D., Evensen, N. M., Martínez, M. L. & De Basabe Delgado, J. Unified equations for the slope, intercept, and standard errors of the best straight line. *Am. J. Phys.* **72**, 367–375 (2004).

[69] Gehrels, W. R. *et al*. Rapid sea-level rise in the North Atlantic Ocean since the first half of the nineteenth century. *The Holocene* **16**, 949–965 (2006).

[70] Saher, M. H. *et al*. Sea-level changes in Iceland and the influence of the North Atlantic Oscillation during the last half millennium. *Quat. Sci. Rev.* **108**, 23–36 (2015).

[71] Kirby, J. R., Garrett, E. & Gehrels, W. R. Holocene relative sea-level changes in northwest Ireland: An empirical test for glacial isostatic adjustment models. *The Holocene* **33**, 926–938 (2023).

[72] Rushby, G. T. *et al*. Testing the mid-Holocene relative sea-level highstand hypothesis in North Wales, UK. *The Holocene* **29**, 1491–1502 (2019).

[73] Barnett, R. L. *et al*. Late Holocene sea-level changes in eastern Québec and potential drivers. *Quat. Sci. Rev.* **203**, 151–169 (2019).

[74] Rossi, V. *et al*. The application of foraminifera to reconstruct the rate of 20th century sea level rise, Morbihan Golfe, Brittany, France. *Quat. Res.* **75**, 24–35 (2011).

[75] Gehrels, W. R. *et al*. A Preindustrial Sea-Level Rise Hotspot Along the Atlantic Coast of North America. *Geophys. Res. Lett.* **47**, e2019GL085814 (2020).

[76] Gehrels, W. R., Belknap, D. F., Black, S. & Newnham, R. M. Rapid sea-level rise in the Gulf of Maine, USA, since AD 1800. *The Holocene* **12**, 383–389 (2002).

[77] Gehrels, W. R. Middle and Late Holocene Sea-Level Changes in Eastern Maine Reconstructed from Foraminiferal Saltmarsh Stratigraphy and AMS 14 C Dates on Basal Peat. *Quat. Res.* **52**, 350–359 (1999).

[78] Shaw, T. A. *et al*. Tectonic influences on late Holocene relative sea levels from the central-eastern Adriatic coast of Croatia. *Quat. Sci. Rev.* **200**, 262–275 (2018).

[79] Kemp, A. C., Whetstine, E. M. & Ridge, J. C. Chronology of late Holocene relative sea-level change in Boston Harbor. *Quat. Sci. Rev.* **346**, 109053 (2024).

[80] Gehrels, W. R. *et al*. Onset of recent rapid sea-level rise in the western Atlantic Ocean. *Quat. Sci. Rev.* **24**, 2083–2100 (2005).

[81] Kemp, A. C. *et al*. Relative sea-level change in Connecticut (USA) during the last 2200 yrs. *Earth Planet. Sci. Lett.* **428**, 217–229 (2015).

[82] Donnelly, J. P., Cleary, P., Newby, P. & Ettinger, R. Coupling instrumental and geological records of sea-level change: Evidence from southern New England of an increase in the rate of sea-level rise in the late 19th century. *Geophys. Res. Lett*. **31**, (2004).

[83] Kemp, A. C. *et al*. Relative sea-level trends in New York City during the past 1500 years. *The Holocene* **27**, 1169–1186 (2017).

[84] Kemp, A. C. *et al*. Sea-level change during the last 2500 years in New Jersey, USA. *Quat. Sci. Rev.* **81**, 90–104 (2013).

[85] Kemp, A. C. & Horton, B. P. Contribution of relative sea-level rise to historical hurricane flooding in New York City. *J. Quat. Sci.* **28**, 537–541 (2013).

[86] Gerlach, M. J. *et al*. Reconstructing Common Era relative sea-level change on the Gulf Coast of Florida. *Mar. Geol.* **390**, 254–269 (2017).

[87] Kemp, A. C. *et al*. Late Holocene sea- and land-level change on the U.S. southeastern Atlantic coast. *Mar. Geol.* **357**, 90–100 (2014).

[88] Yu, H. K. Y. *et al*. The utility of mangrove foraminifera, diatoms, and stable carbon isotope and C/N geochemistry in relative sea-level reconstruction in the Pearl River Delta, China. *Mar. Geol.* **488**, 107610 (2025).

[89] Gehrels, W. R. *et al*. Nineteenth and twentieth century sea-level changes in Tasmania and New Zealand. *Earth Planet. Sci. Lett.* **315–316**, 94–102 (2012).

[90] Gehrels, W. R., Hayward, B. W., Newnham, R. M. & Southall, K. E. A 20th century acceleration of sea-level rise in New Zealand. *Geophys. Res. Lett.* **35**, L02717 (2008).

[91] Garrett, E. *et al*. Drivers of 20th century sea-level change in southern New Zealand determined from proxy and instrumental records. *J. Quat. Sci.* **37**, 1025–1043 (2022).

[92] Grenfell, H. R., Hayward, B. W., Nomura, R. & Sabaa, A. T. A foraminiferal proxy record of 20th century sea-level rise in the Manukau Harbour, New Zealand. *Mar. Freshw. Res.* **63**, 370–384 (2012).

